# Study of Total Antioxidant Activity of Human Serum Blood in the Pathology of Alcoholism

**DOI:** 10.3390/molecules18021811

**Published:** 2013-01-30

**Authors:** Elena I. Korotkova, Wolfhardt Freinbichler, Wolfgang Linert, Elena V. Dorozhko, Mariya V. Bukkel, Evgeniy V. Plotnikov, Olesya A. Voronova

**Affiliations:** 1 Tomsk Polytechnic University, Lenin Avenue 30, 634-050 Tomsk, Russia; 2 Institute of Applied Synthetic Chemistry, Vienna University of Technology, Getreidemarkt 9/163AC, A–1060 Vienna, Austria

**Keywords:** total antioxidant activity, human serum blood, voltammetry, spectrophotometry

## Abstract

The total antioxidant activity (TAA) of human serum blood of patients suffering from alcoholism was tested by cathode voltammetry with a model process of oxygen electroreduction. A known spectrophotometrical method was used for comparison. As results the total antioxidant activity of serum blood of patients with alcoholism was estimated by voltammetry during therapy in hospital. It was shown the TAA of serum blood of patients in pathology before and after treatment is lower than that one of healthy people. However, during the process of 10 days of alcoholism treatment the TAA coefficient increases. The relationship between the coefficient of total antioxidant activity of human serum blood and the stage of treatment was detected.

## 1. Introduction

Alcohol dependence or alcoholism is a substance-related disorder in which an individual is addicted to alcohol either physically or mentally. Alcoholism in a clinical relation has specific criteria for alcohol dependence (tolerance, withdrawal symptoms, use alcohol in larger amounts or for longer periods than intended, *etc.*) and connected with a number of biochemical processes in a human organism [1,2]. In recent studies of the effect of pathogenic factors and drugs (including alcohol) on body’s antioxidant system and the ability to reduce these effects by administering antioxidants is gaining importance [3,4,5,6,7,8,9]. Thus a detailed research of total antioxidant activity (TAA) of human serum blood under physiological and pathological conditions (alcoholism) and TAA possible dependence of antioxidant therapy efficiency is highly prospective.

The antioxidant composition of human serum blood is caused, first of all, by the presence of amino acids, uric acid, ascorbic acid, vitamin E, microelements and intermediate products of metabolism, *etc.* [10,11,12,13,14]. Herewith, TAA of the former mentioned objects is the integral parameter, which characterizes the overall antioxidant potential of all components. These components are situated in a sample not separately but in totality of their interaction in a complex biological system taking into account potential synergism of their interacting antioxidant activity and contribution of minor antioxidants. Sum of separate action of different antioxidants is not equal action of mix of same antioxidant. This antioxidant effect changes during the time. Therefore, we suppose it is more correct to use the term “total antioxidant activity”, rather than capacity.

There are numerous methods of total antioxidant activity determination. But many problems are not clarified, yet [15,16]:

- Lack of comparability of the results of research of antioxidants, carried out by different methods on various model systems;
- Influence of some factors on antioxidant activity of complex biological objects;
- Absence of a single criterion for the estimation of the TAA.

The purpose of the given research is the detection of the TAA of human serum blood under physiological and pathological conditions of alcoholism by cathodic voltammetry. Research on human serum blood under alcoholism pathology conditions was carried out at different stages of treatment. As a method of comparison a well-known spectrophotometrical method had been used. The dependence between parameters of the TAA of human serum blood and stages of therapy was revealed.

## 2. Materials and Methods

### 2.1. Preparation of Serum Sample

In this study we investigated the serum of 50 healthy volunteers (males aged from 20 to 30 years) and 50 male patients (aged from 25 to 45 years) with alcohol dependence syndrome, withdrawal state (according to ICD-10 criteria) treated in Tomsk regional psychiatric hospital. During the experiment, a blood test was performed 3 times in 10 days. Before each experiment, the blood sample was centrifuged for 10 minutes at 2,000 rpm [17].

### 2.2. Detection of Total Antioxidant Activity of Serum Blood by Voltammetry

For the voltammetric study of TAA of human serum blood a AOA (SPE Polyant, Tomsk, Russia) voltammetric analyzer connected with a PC was used in this work. Voltammetric curves were recorded at room temperature in a three-electrode electrochemical cell connecting to the analyzer. A working glassy carbon electrode, a silver–silver chloride electrodes with saturated KCl (Ag|AgCl|KCl), as reference and counter electrodes were used. An open-type cell was used in this investigation.

A working glassy carbon electrode was used with length of 5 mm and diameter of 1.3 mm. It was constant for all measurements. In order to remove residual-adsorbed impurities, the indicator electrode was subjected to 20 voltammetric cycles between 0.0 and −2.0 V at 0.1 Vs^−1^.

As a supporting electrolyte, 0.025 mol·L^−1^ (equimolar mixtures of Na_2_HPO_4_ and KH_2_PO_4_, pH 7.3) phosphate buffer was used. Nanopure water was used for making solutions.

Voltammetric measurements were as follows. A volume of 10 mL phosphate buffer was placed in the electrochemical cell. The measurement of TAA of serum blood involved the recording of voltammograms of the cathodic reduction of oxygen by differential pulse voltammetry without and with addition of 0.2 mL of serum blood into supporting electrolyte under the following conditions: potential rate scan 0.05 Vs^−1^; potential range from 0.0 V to −1.0 V; and amplitude 10 mV. After substance addition and the solution stirring, the potential was scanned negatively, causing oxygen reduction, giving a current wave of oxygen electroreduction (ER O_2_). Its value was proportional to the amount of oxygen in the bulk of the solution. Inhibition of oxygen cathode waves and shear wave potential ER O_2_in the positive region is due to a chemical reaction between antioxidant of serum with active oxygen radicals (in the first place, superoxide anion radicals). Thus, the components of blood serum, is in solution, affect the ER O_2_, showing antioxidant activity. Oxygen concentration was monitored by oxygen analyzer. Based on the ammetric measurements, the concentration of oxygen in phosphate buffer at 25.0 ± 0.5 °C was 2.56 ± 0.05·10^−4^ mol·L^−1^ [18].

### 2.3. Detection of Total Antioxidant Activity of Serum Blood by Spectrophotometry

For the spectrophotometric study of TAA of human serum blood a SF-46 spectrophotometer (LOMO, Leningrad, Russia) was used. Spectrophotometric determination of serum TAA based on the competing reactions of serum and nitroblue tetrazolium (NBT) for superoxide anion radicals of oxygen. In this reaction NBT is reduced with the formation of formazan. In the presence of antioxidants reduction of NBT decreases. Superoxide anion radicals resulted from aerobic interaction of the reduced form of nicotinamide adenine dinucleotide (NAD • H) and phenazine methosulfate (PMS) [19]. The formation of the blue formazan was detected photometrically. The absorbance was measured at 560 nm. To determine the TAA of serum blood 3 mM PMS, 200 mM NAD • H, 50 mM NBT in 0.02 M phosphate buffer of 7.3 pH were used.

## 3. Results and Discussion

Mathematical models were obtained using experimental design methods in order to estimate the significance of influencing factors and to determine the more effective ranges of preparation of serum blood samples [20]. The basic characteristics of the full factor experiment, such as zero level, interval of a variation, top level, bottom level are listed in Table 1. The response function (Y) was chosen as the response of relative change of the ER O_2_ current (equivalent the antioxidant activity): Y = 1 − I/I_0_. The number of experiments is N = 2^2^ = 4. For serum blood a sample mathematical model was obtained [Equation (1)]:

Y = 0.300 + 0.086 X_1_ + 0.139 X_2_ (1)

where *W* [rpm] is speed of centrifugation and *t* [min] is time of centrifugation.

**Table 1:** The basic characteristics of the experiment.

Name of characteristic	X_1_, (*W*)	X_2_, (*t*)
Zero level	1500	6
Interval of a variation	500	4
Top level	2000	10
Bottom level	1000	2

**Figure 1:**
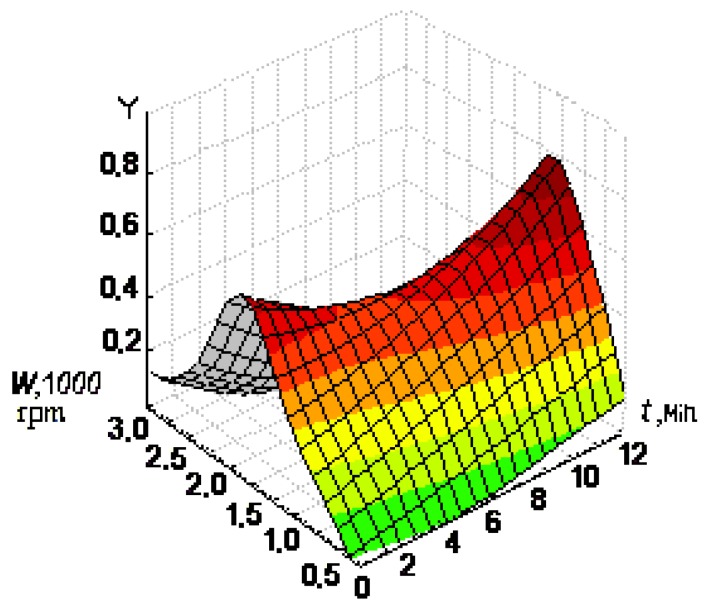
The surfaces of the response of function Y = 1 − I/I_0_ against speed and time of blood centrifugation.

For all samples of serum blood the mathematical model was adequately in the local range of factor space. The second factor (time of centrifugation) had greater influence on the antioxidant activity for all samples. Two-factorial surface of the response is represented in Figure 1. According to this Figure the most effective values of time and speed of centrifugation of serum blood were the following: 10 min at 2,000 rpm.

**Figure 2:**
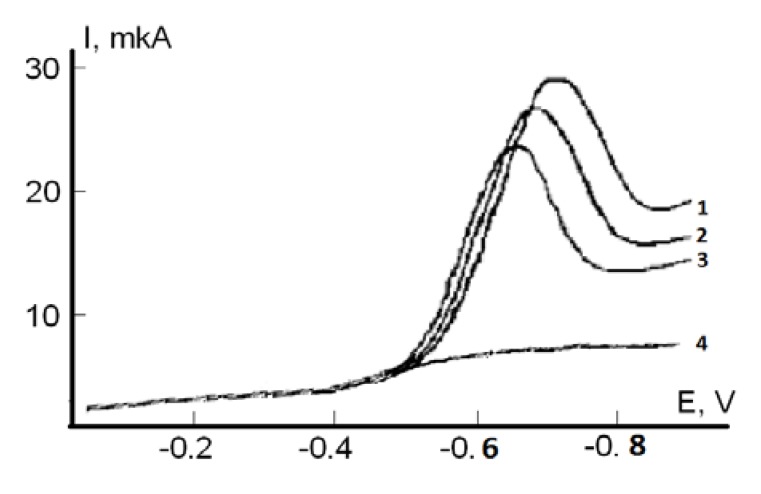
Voltammograms of the ER O_2_ current in phosphate buffer (0.025 M, pH 7.3) on the GCE, without (1) and with 0.2 mL of serum blood at t = 5 min (2), t = 10 min (3). (4) is the residual current without oxygen in the solution.

For the ТAA determination of serum blood by voltammetry the model reaction of the electroreduction of oxygen (ER O_2_) at the working glassy carbon electrode was used. This process is similar to oxygen reduction in tissues. It proceeds at the cathode in several stages with formation of the reactive oxygen species, such as O_2_**^−^** and HO_2_**^−^**. Antioxidants of serum blood are supposed to react with oxygen and its radicals on the GCE surface. At the presence of serum antioxidants the current of ER O_2_ decreases (Figure 2). In this work the coefficient of antioxidant activity of the substances were used in µmol/L·min, which reflects the amount of active oxygen radicals having reacted with the antioxidants for a moment of time, according to the formula:

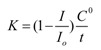

where *C°* [μmol·L^−1^] is the oxygen concentration in solution, *I* is the ER O_2_ current or absorbance coefficient with addition of serum blood sample in the solution, *I_o_* is the limiting ER O_2_ current or absorbance coefficient without the substance in the solution, *t* (min) is time of the interaction between the reactive oxygen species and an antioxidant.

Spectrophotometry is accepted as a comparison method, widely used in biochemical research. In this method formation of the blue formazan of NBT is determined at 560 nm wavelength. In the presence of serum blood antioxidants the rate of NBT reduction decreases. As a result less formazan is produced, which is detected for estimation of TAA of different objects. In Table 2 the results of the comparative detection of the TAA of healthy serum blood (sampling of 50 results) are presented.

**Table 2:** Values of the TAA of human serum blood under physiological conditions, calculated by the kinetic coefficient, measured by two various methods.

Number of sample (serum of healthy donors)	*К*, µmol/L·min		∆, %
	Spectrophotometry	Voltammetry	
1	0.83 ± 0.04	0.85 ± 0.05	2.30
2	0.95 ± 0.03	1.01 ± 0.02	6.12
3	0.93 ± 0.03	0.97 ± 0.03	4.21
4	0.87 ± 0.02	0.91 ± 0.06	4.49
5	0.92 ± 0.05	1.00 ± 0.04	8.33

As it can be seen in Table 2 the results of both methods correlate with each other, whereby the spectrophotometric method showed slightly understated results. However, the voltammetric detection of the TAA of serum blood appeared to be more sensitive and required less reactant for carrying out the analysis. In Table 3 the results of serum blood TAA measurements of patients with alcoholism are presented.

**Figure 3:**
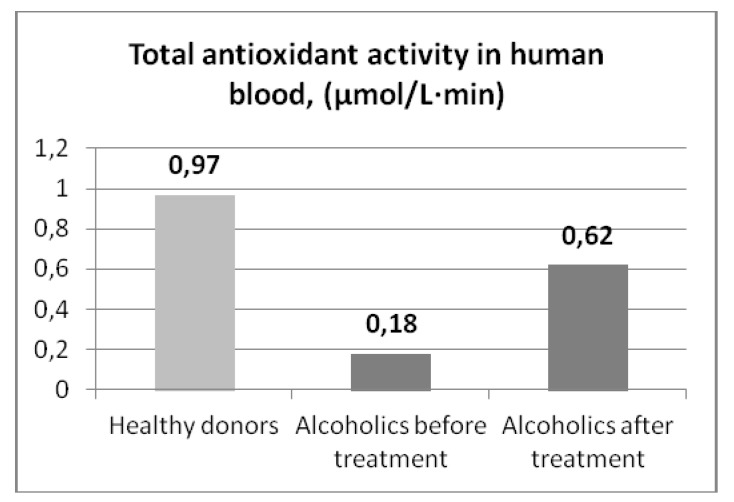
Total antioxidant activity of serum blood (patients with alcoholism).

As it can be seen from Table 3, TAA coefficients change from patient to patient at the same stage of treatment. This probably depends on the physical health of the patient and the initial state. In Figure 3 results of the relationships of the TAA of human serum blood of patients with alcoholism and the stage of treatment are presented. According to Figure 3 the low values of the TAA of serum blood of patients at arrival in the hospital (К = 0.18 ± 0.04 µmol/L·min), apparently related with a general depression of the organism after a long period of alcohol abuse. Reduction of serum TAA after a series of intravenous infusions is probably due to the increase in overall hydration.

**Table 3:** Values of the TAA of serum blood of patients with alcoholism at different stages of treatment (n = 10).

No.	*К*, µmol/L·min		
	Arrival in the hospital	The period of detoxication (begining of treatment)	After 10 days of treatment in the hospital
1	0.11 ± 0.04	0.08 ± 0.02	0.82 ± 0.03
2	0.21 ± 0.03	0.11 ± 0.04	0.81 ± 0.05
3	0.02 ± 0.01	0.02 ± 0.01	0.15 ± 0.04
4	0.41 ± 0.03	0.12 ± 0.04	0.72 ± 0.04
5	0.25 ± 0.05	0.15 ± 0.02	0.63 ± 0.03
6	0.32 ± 0.02	0.11 ± 0.03	0.54 ± 0.06
7	0.02 ± 0.01	0.02 ± 0.01	0.44 ± 0.04
8	0.32 ± 0.05	0.13 ± 0.05	0.65 ± 0.05
8	0.21 ± 0.04	0.13 ± 0.04	0.75 ± 0.04
10	0.04 ± 0.02	0.04 ± 0.01	0.35 ± 0.03

At the treatment’s closing stage (days 6–9 of staying in the hospital) an increase of the TAA of serum blood of all patients was observed. It is result of improvement of the somatic condition of the patients during the therapy. Probably, it is related with the increase of natural antioxidant substances amount (ascorbic acid, tocopherol) in the blood of the patients [21]. It should be noted, that in the course of treatment patients received antioxidants in remedies (vitamins C, E) and quality food, which significantly contributes to the restoration of blood antioxidant system, including through the accumulation of low molecular weight antioxidant components. However, it is necessary to note, that even at the final stage of therapy the average level of the TAA of serum blood (0.62 ± 0.04 µmol/L·min) remained significantly lower than the similar indicator of healthy donors (0.97 ± 0.1 µmol/L·min). So, wider use of antioxidants in therapy of alcoholism and continuation of research of the given direction is justified.

## 4. Conclusions

Exhaustion of antioxidant systems of the patients with alcoholism and TAA relationships with the stage of treatment were detected. Findings suggest the activation of the formation of free radicals in the body of patients with alcoholism. Based on the analysis, we can conclude that the rate of the total antioxidant activity in the blood serum is a general non-specific marker of pathological changes in the patient with alcoholism. After treatment, TAA coefficient increase partially, which suggests the necessity for special antioxidant therapy in this condition. TAA measurements allow one to indirectly evaluate the effectiveness of treatments.
